# First report on molecular characteristics and risk factor analysis of *Ehrlichia canis* in dogs in Khon Kaen, Thailand

**DOI:** 10.14202/vetworld.2022.232-238

**Published:** 2022-01-31

**Authors:** Thongphet Mitpasa, Biethee Rani Sarker, Arayaporn Macotpet, Pattara-Anong Bupata, Somboon Sangmaneedet, Weerapol Taweenan

**Affiliations:** 1 Department of Pathobiology, Faculty of Veterinary Medicine, Khon Kaen University, Khon Kaen 40002, Thailand; 2 Department of Medicine, Faculty of Veterinary Medicine, Khon Kaen University, Khon Kaen 40002, Thailand; 3 Veterinary Teaching Hospital, Faculty of Veterinary Medicine, Khon Kaen University, Khon Kaen 40002, Thailand.

**Keywords:** *Ehrlichia canis*, molecular characteristics, nested polymerase chain reaction, phylogenetic analysis, Thailand

## Abstract

**Background and Aim:**

*Ehrlichia canis* is a well-known cause of both anemia and thrombocytopenia in dogs. There are insufficient epidemiological data on this blood parasite in Thailand and the association of infections with hematological abnormalities. This study aimed to analyze the molecular characteristics and to identify *E. canis* as well as the risk factors associated with *E. canis* infection in dogs in Khon Kaen, Thailand.

**Materials and Methods:**

Blood samples from 126 dogs that visited animal clinics were subjected to molecular detection using nested polymerase chain reaction for *E. canis* *16S rRNA* gene. The risk factors and hematological profiles associated with the infection were analyzed using the logistic regression test in program SPSS version 19.

**Results:**

Forty-one dogs were infected, indicating a 32.5% molecular infection rate of *E. canis*. The factors significantly associated with *E. canis* infection include animal housing status, low packed cell volume, low red blood cell count, and low platelets (p<0.05). Ten positive samples were amplified, sequenced, and phylogenetically analyzed. Sequence and phylogenetic analysis confirmed the current ten samples as *E. canis* compared with reference sequences in GenBank, using the BLAST program hosted by NCBI, which showed 99.74-100% similarity.

**Conclusion:**

This study provided the first data of infection rate of *E. canis* using nested PCR and molecular characteristics of *E. canis* in randomly selected domestic dogs in Khon Kaen, Thailand.

## Introduction

Canine monocytic ehrlichiosis (CME) is an infectious disease caused by *Ehrlichia canis* [1,2]. *E. canis*, an obligate intracellular gram-negative bacterium, belongs to the order *Rickettsiales* [3]. Notably, the disease occurs in animals and humans [4-6]. The brown dog tick, *Rhipicephalus sanguineus*, is responsible for transmitting *E. canis* [7,8]. Ehrlichiosis is a febrile sickness associated with leukopenia, thrombocytopenia, and anemia in dogs [9]. *E. canis* infection in dogs induces various clinical presentations: Acute phase, subclinical phase, and chronic phases. It also causes human infection [4,10]. *E. canis* occurs globally, especially in tropical and subtropical regions [7,11-13].

Khon Kaen is a city located northeast of Thailand. Although a couple of reports have shown *E. canis* infection, a study was conducted in a small-scale sub-district area using the conventional polymerase chain reaction (PCR) technique [14]. Another study used multiplex PCR to detect *E. canis* in left-over blood samples negative for *E. canis* microscopically in a clinic [15]. This study covered private animal clinics on a large scale in the city municipality area and the Veterinary Teaching Hospital, Khon Kaen University (KKU), located in the Muang district, Khon Kaen. Nested PCR was conducted rather than multiplex or conventional PCR. Furthermore, this parasite’s molecular characteristics and phylogenetic analysis have never been described in the northeast of Thailand.

Consequently, this study aimed to investigate the molecular characteristics of *E. canis* infection in the city municipality area of Khon Kaen and to assess the association between infection and various factors, including the age of the dogs, rearing status, tick infestation status, packed cell volume (PCV) level, red blood cell (RBC) count, white blood cell (WBC) count, and platelet count. It is hoped that the results will assist in controlling and preventing *E. canis* infection in dogs and facilitate a decline in disease transmission to humans.

## Materials and Methods

### Ethical approval

The study was reviewed and approved by the Institutional Animal Care and Use Committee of KKU, No IACUC-KKU-7/64. The written consent from owners of animals participating in the research was obtained.

### Study period, area, and sample collection

**Figure-1:**
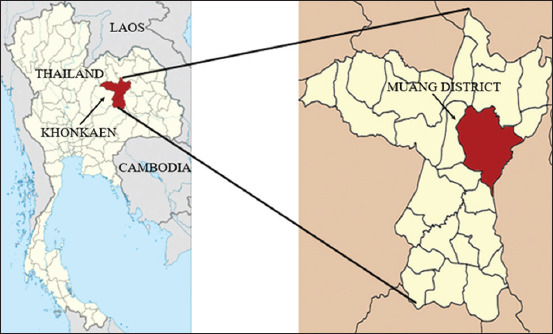
Map of Khon Kaen Province, Thailand, where blood samples were collected [Source: https://doi.org/10.1186/s12889-018-5871-1].

From June to December 2020, 126 blood samples were randomly collected from dogs visiting seven private animal clinics and the KKU Veterinary Teaching Hospital in the city municipality area, Muang district, Khon Kaen, Northeast Thailand (Figure-1). The sample size calculation was based on 36.73% prevalence of *E.canis* infection from the previous study in Buriram, the province located near Khon Kaen [16], with a 10% error. Dogs with or without any clinical signs were randomly selected in this study. Three milliliters of blood samples were collected from the cephalic or saphenous vein, filled in EDTA-anticoagulant (BD Vacutainer®, New Jersey, USA) tubes, and kept in an icebox (4-8°C) before being sent to the laboratory within eight hours at the Faculty of Veterinary Medicine, KKU, Khon Kaen, for further investigation.

### Risk factor and hematological analysis

Some information, including dog age (≤1 year and >1 year), the duration of tick infestation (no ticks, <1 month, 1-6 months, and >6 months), and animal housing status (indoor, confined to grounds, and free-roaming), were collected by interviewing the pet owners.

The levels of the PCV, RBC count, WBC count, and platelet count of all samples were examined by the Vet Central Lab, Khon Kaen. Anemia severity on the basis PCV was classified as follows: Normal (PCV >37%), mild (30-37%), moderate (20-29%), and severe (<20%) [17]. For platelet count, four criteria for the interpretation of the results were applied. The criteria were as follows: Non-thrombocytopenia (platelets ≥200,000 cell/μL), mild (platelets 150,000-199,000 cell/μL), moderate (platelet 100,000-149,000 cell/μL), and severe thrombocytopenia (platelet <100,000 cell/μL) [17]. The RBC was classified as non-anemic or anemic following the Vet Central Lab, Khon Kaen, 2020, with a normal RBC range of 5-9×10^6^ cells/mm^3^. The WBC was classified as non-leukopenia or leukopenia according to the Vet Central Lab, Khon Kaen, 2020, with a normal WBC range of 6000-17,000 cells/mm^3^.

### Molecular detection

DNA was extracted from blood samples using the GF-1 blood DNA kit (Vivantis, Selangor Ehsan, Malaysia), according to the manufacturer’s instructions. The nested PCR protocol followed the previous study in amplifying the *16S rRNA* gene. The molecular-grade water was used as the negative control. The first reaction using primers ECC (5’ AGAACGAACGCTGGCGGCAAGCC 3’) and ECB (5’ CGTATTACCGCGGCTGCTGGCA 3’) and the second reaction using primers CANIS (5’ CAATTATTTATAGCCTCTGGCTATAGGA 3’) and HE3 (5’-TATAGGTACCGTCATTATCTTCCCTAT 3’), yielded 478 and 389 bp amplicons, respectively [18]. The PCR products were sequenced using primer CANIS and HE3 (BTSeq™ Barcode-Tagged Sequencing; Celemics, Seoul, South Korea). The current sequences were compared with the sequences in GenBank, using the BLAST program hosted by NCBI, National Institutes of Health, USA (http://www.ncbi.nlm.nih.gov) to evaluate the similarity before being aligned using BioEdit version 7 software (developed by Tom Hall, North Carolina State University) [19]. The phylogenetic tree was constructed using the neighbor-joining method with 500 replicates for bootstrap analysis, MEGA version 7 software (Molecular Evolutionary Genetics Analysis software available at https://www.megasoftware.net/) [20].

### Statistical analysis

The risk factors, RBC, WBC, platelets, and PCV, were analyzed in relation to the infection. The relationship between risk factors and prevalence was analyzed using the logistic regression test with p<0.05 in SPSS software v 19.0 (IBM Corp., NY, USA).

## Results

### Molecular detection of *E. canis* and association analysis

**Figure-2:**
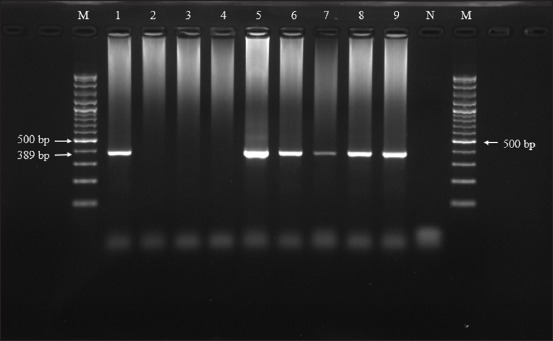
Gel electrophoresis results for the nested polymerase chain reaction of *E. canis*, Lanes 1, 5, 6, 7, 8, 9=Positive samples; Lanes 2, 3, 4=Negative samples; N=Negative control; M=100bp marker.

The PCR results showed that from the 126 samples, 41 samples (32.5%, 95% CI: 23.6-41.46%) were positive and 85 samples (67.5%) were negative (Figure-2).

According to the statistical analysis of risk factors, there was an association between animal housing status and *E. canis* infection (p<0.01), while age and tick infestation was not associated (p>0.05). The free-roaming dogs had a higher *E. canis* infection rate of 53.3% (24/45) than the other groups (Table-1).

**Table-1:** Association between risk factors and *Ehrlichia canis* infection.

Variable	% Prevalence (No. of positive/No. of samples)	Odds ratio (95% CI)	Adjusted odds ratio (95% CI)	p-value
Age (year)				
≤1	37.5 (12/32)	1	1	0.385
>1	30.9 (29/94)	0.74 (0.32-1.72)	0.66 (0.26-1.68)	
Tick infestation				
No tick	7.1 (1/14)	1	1	0.196
<1 month	35.3 (12/34)	7.09 (0.82-61.00)	3.02 (0.52-17.78)	0.220
1-6 months	48.6 (18/37)	12.32 (1.46-104.01)	5.00 (0.86-29.19)	0.074
>6 months	24.4 (10/41)	4.19 (0.48-36.19)	2.04 (0.34-12.24)	0.436
Animal housing status				
Indoor	10 (1/10)	1	1	0.011
Confined to grounds	22.54 (16/71)	2.62 (0.31-22.25)	1.95 (0.22-17.23)	0.553
Free roaming	53.3 (24/45)	10.29 (1.20-88.07)	6.59 (0.72-59.49)	0.095

CI=Confidence interval

Among the 126 dogs, 7.6% (6/79) of the non-anemic dogs were found positive for *E. canis*, while 43.8% (7/16) were positive in the mildly anemic group. Moreover, the moderately and severely anemic dogs were found positive in 86.7% (13/15) and 93.8% (15/16), respectively. Results from the logistic regression test showed a statistically significant relationship between levels of PCV and the number of *E. canis* positive dogs (p<0.05). According to the WBC, 32.2% (38/118) of positive cases were found in the non-leukopenia group, whereas 37.5% (3/8) of the positive cases were found in the leukopenia group. Logistic regression tests showed no association between WBC and *E. canis* infection (p>0.05). The results indicated that 19.2% (19/99) of non-anemic dogs and 81.5% (22/27) of anemic dogs were positive for *E. canis* infection. Logistic regression test showed a significant relationship between RBC and *E. canis* infection (p<0.05). According to platelet count analysis, 22.9% (19/83), 50% (8/16), 35.7% (5/14), and 69.2% (9/13) were found positive in the non-thrombocytopenia, mild thrombocytopenia, moderate thrombocytopenia, and severe thrombocytopenia groups, respectively. Results from the logistic regression test showed a statistically significant relationship between platelet levels and *E. canis* infection (p<0.05) (Table-2).

**Table-2:** Association between hematology and *Ehrlichia canis* infection.

Variable	% Prevalence (No. of positive/No. of samples)	Odds ratio (95% Confidence interval)	p-value
Platelets (cells/µL)			
≥200,000	22.9 (19/83)	1	
150,000-199,000	50.0 (8/16))	3.37 (1.12-10.18)	0.031
100,000-149,000	35.7 (5/14)	1.87 (0.56-6.26)	0.309
<100,000	69.2 (9/13)	7.57 (2.10-27.38)	0.002
Packed cell volume (%)			
≥37	7.6 (6/79)	1	
30-36	43.8 (7/16)	9.46 (2.60-34.44)	0.001
20-29	86.7 (13/15)	79.08 (14.37-435.33)	0.001
<20	93.8 (15/16)	182.50 (20.45-1628.56)	0.001
Red blood cell			
Non-anemia	19.2 (19/99)	1	0.001
Anemia	81.5 (22/27)	18.53 (6.21-55.23)	
White blood cell			
Normal	32.2 (38/118)	1	0.757
Abnormal	37.5 (3/8)	1.26 (0.28-5.56)	

### Sequence analysis

**Figure-3:**
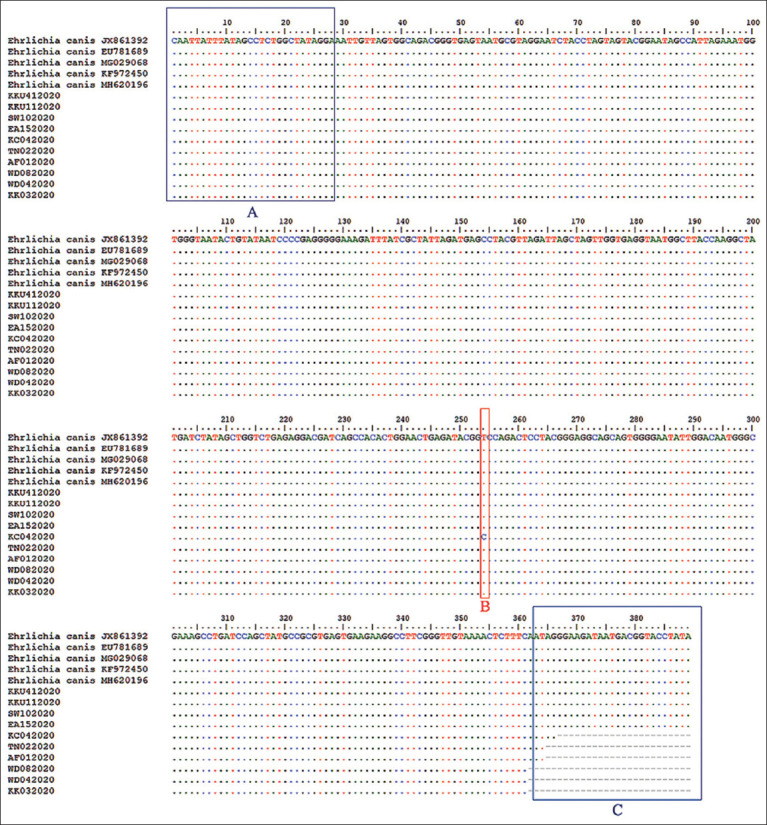
Alignment of the sequences obtained from *16S rRNA* gene of *Ehrlichia canis* used in this study and other *E. canis* from GenBank. The dot (.) and dash (­­) denote the identical nucleotides and the absence of nucleotides, respectively. Box A and C denote forward and reverse primers, respectively. Box B denotes a different base at position 254.

Among the 41 PCR-positive samples, 10 were randomly selected and sent to the company (CELEMICS, Seoul, South Korea) for sequencing. The sequence IDs of our samples were given as follows: SW102020, KK032020, WD042020, WD082020, AF012020, KKU112020, KKU412020, TN022020, EA152020, and KC042020. The results were similar to *E. canis* strains from Texas, Tunisia, Mexico, India, and Brazil (GenBank accession no. MH620196, EU781689, MG029068, JX861392, and KF972450) with similarities of 99.74–100%, respectively. However, one sample, KC042020, from this study showed a different base at position 254 (Figure-3).

**Figure-4:**
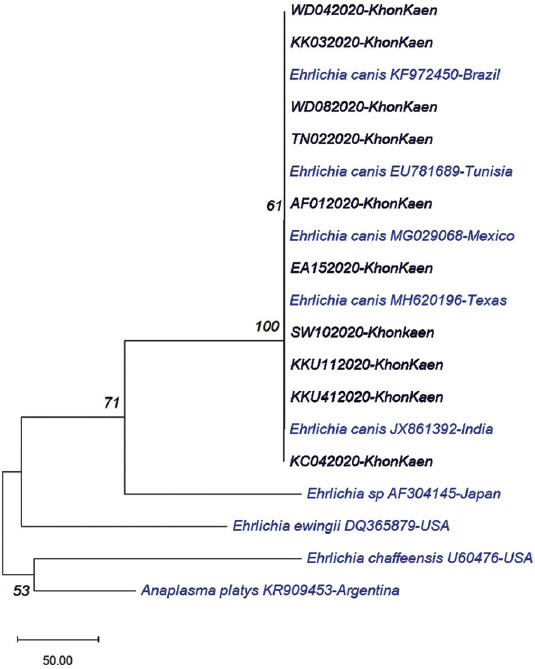
Phylogenetic analysis of 10 *Ehrlichia canis* samples identified in Khon Kaen and other *E. canis* in GenBank based on *16S rRNA* gene. The phylogenetic tree was achieved by the neighbor-joining method. The numbers at the nodes are the proportions of 500 bootstrap with Kimura 2-parameter model. The current samples are highlighted in bold.

The phylogenetic tree was constructed using the neighbor-joining method in the MEGA (version7) program. *Ehrlichia* spp., *Ehrlichia ewingii, Ehrlichia chaffeensis*, and *Anaplasma platys* sequences from the GenBank were compared as the out-group. The results revealed that the samples were closely related to *E. canis* from GenBank accession no. MH620196, EU781689, MG029068, KF972450, and JX861392 (Figure-4).

## Discussion

This study observed the infection rate and molecular characteristics of *E. canis* in dogs in Khon Kaen. The association between *E. canis* infection and risk factors, including the levels of PCV, WBC, RBC, and platelet count was evaluated. The infection rate (32.5%) was similar to the previous reports in some other countries. For example, *E. canis* prevalence was found to be 21.8% in Cambodia [21], 28% in Pakistan [22], and 47.1% in Malaysia [23]. In Colombia, the prevalence was 40.6% [24], whereas *E. canis* infection prevalence was as low as 6.3% in Northeast Algeria [25]. Different temperatures in different countries influence the tick population, which may cause several *E. canis* infection prevalence.

In Thailand, several studies have reported the prevalence of *E. canis* infection from different provinces, including 21.5% in Mahasarakham [26], 25% in Kalasin [17], 36.73% in Buriram [16], and 65.12% in Bangkok and Samutraprakarn [27]. In Khon Kaen, where this research was conducted, other studies were previously reported using different methodologies [14,15]. For example, one study used multiplex PCR to detect infection in left-over blood samples from animal hospitals, which were primarily negative following microscopic examination and found 33.75% prevalence of *E. canis* infection [15]. However, this study collected random blood samples from hospitalized dogs. Contrastly, another study that collected samples from Phra Lap Sub-district, Muang District, Khon Kaen, used conventional PCR to examine *E. canis* and showed a low infection prevalence of 3% [14].

CME occurrence in dogs in Khon Kaen Province was shown to be associated with animal housing status (p=0.001). Dogs living in indoor areas were at less risk of *E. canis* infection, probably related to low tick infestation [28]. Another report suggested that dogs living in outdoor areas were at higher risk of *E. canis* infection (Odds Ratio 2.3) than dogs living indoors [29]. In this study, tick infestation was not associated with *E. canis* infection (p=0.196). The result agreed with a previous study showing that ticks were not considered a risk factor for *E. canis* positivity [20]. However, a previous study in Mexico showed 72% prevalence of *E. canis* infection in dogs infested with tick and 54% in dogs without tick infestation [30].

Furthermore, *E. canis* infection occurs mainly during the warm season compared to the rainy and winter [31]. The differences in climatic conditions have been found to be important factors influencing the population dynamics of ticks in a particular region, resulting in a variable prevalence pattern of CME [32]. The dog’s age was not associated with *E. canis* infection (p=0.488), and this was also supported by the study of *E. canis* infected dogs in three districts in Punjab of Pakistan [22]. However, one previous report found that dogs younger than 6 months had more *E. canis* infection than older dogs [31]. However, the gender of animals was not analyzed in the current research since several studies have revealed that it was not associated with the infection [22,33,34].

PCV values showed a significant association with *E. canis* infection (p=0.001). This result agrees with the previous reports [9,17,18]. The current results show that PCV values of <20% were associated with more *E. canis* infection (93.8%). This study had similar findings to a previous report [35]. In addition, platelet count was associated with *E. canis* infection (p=0.002), as supported by the previous reports [36,37]. This study showed that dogs with platelet counts <100,000 platelets/mL had a higher infection rate (69.2%) than other groups (Table-2). Moreover, RBC count was associated with *E. canis* infection (p=0.001) [9]. Similar to this previous study, the present study showed that anemic dogs had an increased *E. canis* infection rate (81.2%) [38]. In addition, WBC count was not associated with *E. canis* infection (p=0.757), and this is in agreement with a previous report [36].

According to the BLAST investigation, the partial sequences of the *16S rRNA* gene of *E. canis* in dogs in Khon Kaen Province were more than 99% identical to genotypes in GenBank isolated from Texas, Tunisia, Mexico, India, and Brazil. The phylogenetic tree and sequence alignments showed low diversity in *E. canis* strains, similar to the previous reports [39,40]. However, it would be interesting to examine longer *16S rRNA* sequences of *E. canis* strains to detect genetic diversity in Thai dogs. Furthermore, no heterogeneity was observed among the *E. canis* groups using the *16S rRNA* gene. However, the distinct sequence was found to have a 99% similarity to the *Ehrlichia* sequence reported from China [41].

A previous study from Bangkok showed that the *E. canis* strains were linked with multiple connected branches, and little genetic diversity was observed, suggesting slow and homogeneous evolution [42]. This study supports this concept as *E. canis* strains were also joined with multiple connecting branches. The phylogenetic tree and sequence alignments showed low diversity in *E. canis* strains [43]. The extremely low polymorphism exhibited among the *E. canis* strains[40], suggests a common origin with no distant divergence in the dendrogram showing the genetic relationship between the *16S rRNA* sequences of different *Ehrlichia* spp. isolates.

## Conclusion

This study provided the first data on the infection rate of *E. canis* using nested PCR and molecular characteristics of *E. canis* in domestic dogs in Muang district, Khon Kaen Province, Thailand. The phylogenetic trees display a high similarity of the current *E. canis* and other sequences in GenBank. Conclusively, 32.5% of dogs were infected with *E. canis*. Dogs living outdoor (p<0.05) were associated with infection, whereas age and tick infestation was not related. Furthermore, low PCV levels, low platelets, and low RBC were associated with *E. canis* infection. This survey provided preliminary information on the prevalence and factors associated with CME, which can be helpful in developing control and prevention program. Animal housing indoors or confined areas should be concerned, and pet owners should consider controlling tick infestation.

## Authors’ Contributions

WT: Conceptualization, formal analysis, project administration, and drafted and revised the manuscript. TM and WT: Methodology, data curation, visualization. TM, BRS, AM, and PB: Investigation. TM: Software and the first draft of the manuscript. SS: Supervision. WT and SS: Validation. All authors read and approved the final manuscript.
